# Investigation of Carers’ Perspectives of Dementia Misconceptions on Twitter: Focus Group Study

**DOI:** 10.2196/30388

**Published:** 2022-01-24

**Authors:** Georgie Hudson, Sonja M Jansli, Sinan Erturk, Daniel Morris, Clarissa M Odoi, Angela Clayton-Turner, Vanessa Bray, Gill Yourston, Doreen Clouden, David Proudfoot, Andrew Cornwall, Claire Waldron, Til Wykes, Sagar Jilka

**Affiliations:** 1 Institute of Psychiatry, Psychology, and Neuroscience King's College London London United Kingdom; 2 South London and Maudsley NHS Foundation Trust London United Kingdom; 3 Warwick Medical School University of Warwick Coventry United Kingdom

**Keywords:** patient and public involvement, dementia, co-production, misconceptions, stigma, Twitter, social media, Alzheimer’s Disease

## Abstract

**Background:**

Dementia misconceptions on social media are common, with negative effects on people with the condition, their carers, and those who know them. This study codeveloped a thematic framework with carers to understand the forms these misconceptions take on Twitter.

**Objective:**

The aim of this study is to identify and analyze types of dementia conversations on Twitter using participatory methods.

**Methods:**

A total of 3 focus groups with dementia carers were held to develop a framework of dementia misconceptions based on their experiences. Dementia-related tweets were collected from Twitter’s official application programming interface using neutral and negative search terms defined by the literature and by carers (N=48,211). A sample of these tweets was selected with equal numbers of neutral and negative words (n=1497), which was validated in individual ratings by carers. We then used the framework to analyze, in detail, a sample of carer-rated negative tweets (n=863).

**Results:**

A total of 25.94% (12,507/48,211) of our tweet corpus contained negative search terms about dementia. The carers’ framework had 3 negative and 3 neutral categories. Our thematic analysis of carer-rated negative tweets found 9 themes, including the use of weaponizing language to insult politicians (469/863, 54.3%), using dehumanizing or outdated words or statements about members of the public (n=143, 16.6%), unfounded claims about the cures or causes of dementia (n=11, 1.3%), or providing armchair diagnoses of dementia (n=21, 2.4%).

**Conclusions:**

This is the first study to use participatory methods to develop a framework that identifies dementia misconceptions on Twitter. We show that misconceptions and stigmatizing language are not rare. They manifest through minimizing and underestimating language. Web-based campaigns aiming to reduce discrimination and stigma about dementia could target those who use negative vocabulary and reduce the misconceptions that are being propagated, thus improving general awareness.

## Introduction

The World Alzheimer’s Report [1] highlighted the damaging negative attitudes about dementia, “as the resulting shame, guilt, hopelessness, and social exclusion, lead to delayed diagnosis [2], inability to cope, decreased quality of life [3] and increased burden of dementia (eg, excess disability [4]).” These issues also extend to friends, family, and caregivers of individuals with dementia, as they become the target of stigmatizing views “by association” [5]. Myths and misconceptions about dementia can also lead to a lack of open communication [6]. The use of devaluing words, such as “demented” is especially common on social media platforms such as Twitter [7], and many tweets contain language that ridicules the disease and therefore perpetuates the associated stigma [8]. Twitter is a popular international social media service, with the vast majority of tweets being public and thus reaching a wide audience [9]. It also has a high prevalence of stigma towards dementia [10] and therefore lends itself to investigations into misconceptions.

Given the multiple negative consequences, it is surprising that little is known about the prevalence of public misconceptions on social media. Improving the overall knowledge base for dementia, especially a detailed understanding of the types of misconceptions, can provide a baseline from which to challenge misconceptions and stigma [11]. Although previous work examined types of dementia-related conversations on Twitter from the researchers’ perspective [12-14], none have taken the views of those with lived experience into account to understand misconceptions. We argue that involvement through participatory methods is the first step to understanding the social media content that perpetuates dementia misconceptions and stigma. This study overcomes this gap by codeveloping a framework with carers to understand, in detail, the forms of dementia misconceptions on Twitter.

## Methods

### Design

**Figure 1:**
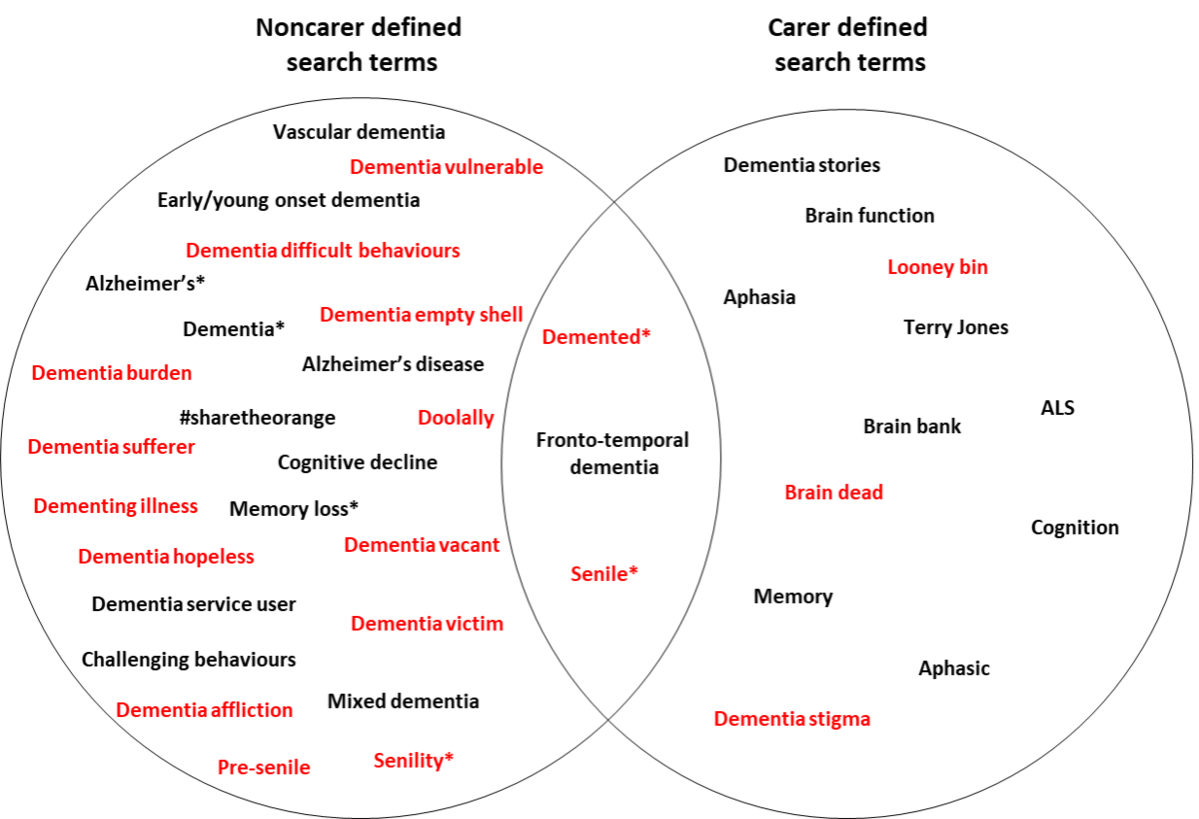
Neutral (black) and negative (red) search terms, as defined by carers and noncarers (eg, through researchers’ own Twitter search, or the literature). Words with an asterisk were taken from Oscar et al [8].

This was a mixed methods study using participatory methods [15,16] with carers of people with dementia. We held 3 focus groups with carers to identify search terms for data collection and generated an initial framework of misconceptions. Search terms (Figure 1) from carers, the literature, researchers’ Twitter searches, and dementia awareness campaigns were used to extract tweets (described next in “Tweet Collection and Screening”) and carers’ feedback iteratively refined the framework. Carers then individually categorized tweets into the framework and their interrater reliability was examined. The final framework was used by service user researchers (researchers with lived experience of using mental health services) to analyze tweets that carers categorized as negative.

### Participants and Recruitment

Participants were recruited if they had experience caring for someone with a diagnosis of dementia. We recruited from (1) a research advisory group, MALADY [17], made up of dementia carers, and (2) Join Dementia Research, a United Kingdom–wide web-based platform hosted by the National Institute for Health Research (NIHR). Participants were included if they were at least 18 years old and were dementia carers who could give capacity to consent. A total of 7 carers were recruited and invited to take part in as many of the research activities as possible.

#### Patient Involvement 

Dementia carers were involved as participants and were involved in the design, project management, and data analysis for this paper; they are also authors of this paper.

### Tweet Collection and Screening

**Figure 2:**
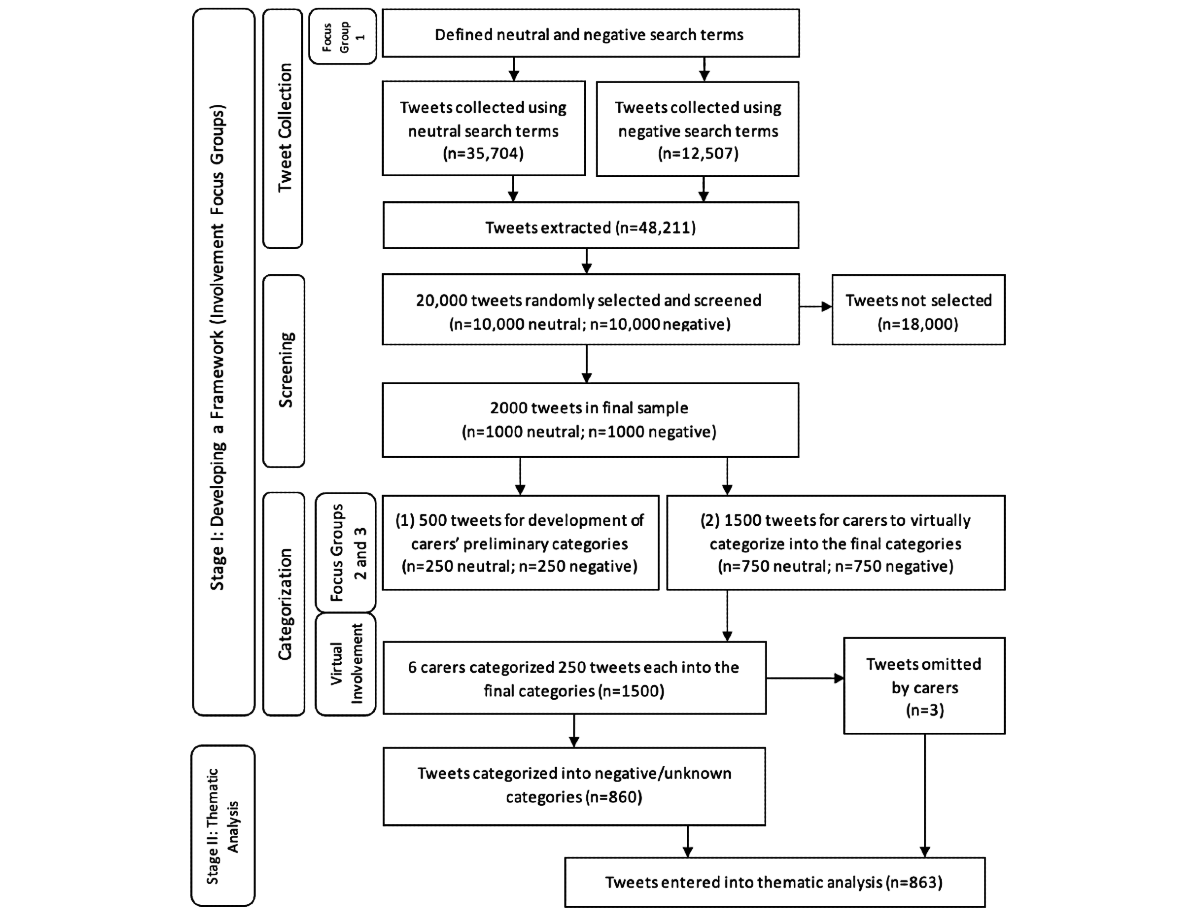
Tweet extraction and categorization, outlining the number of tweets extracted, screened, not selected, and categorized by carers.

Publicly available tweets originating from across the world were extracted in real time between February 4 and 7, 2020, using Twitter’s streaming application programming interface (API). The connection to Twitter’s API was made via Python’s open source Tweepy library (Python Software Foundation). Tweets were captured if they contained any occurrence of the English dementia search terms identified by carers, those previously cited by Oscar et al [8], or words identified in tweets from patient advocacy groups or awareness campaigns. Most words were directly associated with dementia (see Figure 1), but some words or phrases not specific to dementia were also included because carers thought they related to negative aspects of dementia. Through a discussion with carers, there was a lack of agreement on what differentiated a positive term from a neutral term; therefore, we asked the carers to simply categorize words as either negative or neutral (which included positive) search terms. All search terms were then defined by carers as negative or neutral. The stages of analysis are shown in Figure 2. A total of 48,211 tweets were collected, 35,704 using neutral search terms and 12,507 using negative search terms (see Multimedia Appendix 1 for a breakdown of tweet collection). To manage this data set, 10,000 neutral and 10,000 negative tweets were randomly selected. From these 20,000, we selected 2000 tweets (1000 negative and 1000 neutral) that met the following criteria: (1) written in English, (2) made clear reference to dementia, (3) had a comprehensible meaning (ie, not a Uniform Resource Locator [URL] or a random string of words generated by a bot), and (4) were neutral or negative. These 2000 tweets were given to carers to carry out 2 tasks. First, carers coded a subsample of tweets (n=500) and subsequently refined their initial framework. Then, carers were given the remaining 1500 tweets to code into the final categories (see Figure 2 for an overview). This number and the types of tweets were defined through discussion with the carers on the burden tweet rating would place on them.

### Ethical Approval and Procedure

The study was granted ethical approval from the King’s College London Psychiatry, Nursing and Midwifery Research Ethics Committee on December 4, 2019 (HR-19/20-14565). The procedure consisted of 2 stages.

#### Stage I: Developing a Framework (Involvement Focus Groups)

Carer involvement was spread across 3 focus groups. Each group followed a prespecified structure, which incorporated strategies to facilitate coproduction [18]. These focus groups took place in person (Multimedia Appendix 2).

The process of building the framework fell into 3 steps. In step 1, focus group members (n=4) described their experiences, browsed Twitter in order to generate a list of dementia search terms, and categorized the search terms as either negative or neutral (Figure 1). In step 2, the researchers generated an initial framework. In both focus groups 2 and 3, carers (n=5) categorized 250 tweets randomly selected from the sample of 2000 into themes, refined them, and created new ones (Multimedia Appendix 3). The final framework included all their feedback. Finally in step 3, 6 carers were each emailed a different set of 250 randomly collected tweets, and they categorized their set of tweets independently into the final framework. Each tweet was coded only into one category. This step was carried out via email due to the COVID-19 pandemic. Interrater reliability was assessed by each of the 6 carers categorizing 50 rated tweets (10 tweets from each of the 5 other carers). The total number of tweets used for the assessment of interrater reliability was 300.

#### Stage II: Qualitative Analysis of Tweets

We focused our analysis on the tweets that carers categorized as negative and tweets that carers were unsure of or missed. See Figure 2 for a breakdown of tweet extraction and categorization.

### Data Analysis

An interrater reliability analysis using the kappa statistic was performed to determine consistency among carer raters in the final development of the framework. We report the average kappa score across all carers and the range.

Service user researchers carried out a thematic framework approach [19] for the qualitative analysis of the tweets. All tweets that the carers categorized into negative framework categories as well as those categorized as “other” or “I don’t know,” and the tweets the carers omitted, were thematically analyzed as one data set, employing an inductive, holistic methodology. This process involved (1) familiarization with the data by reading through the tweets, (2) coding the tweets, (3) combining the relevant tweets together, (4) examining the codes (ie, the framework categories) to identify themes, (5) reviewing and refining the themes, and (6) defining and naming the themes. Two researchers independently conducted this analysis using NVivo 12 for Windows (QSR International), identifying themes and subthemes within the framework created by the carers. The researchers compared their coding, and any discrepancies were resolved through discussion between the researchers. A guidance document (Multimedia Appendix 4) was written with the criteria used by the 2 researchers to categorize the tweets, which was used by a third researcher to ensure consistency of the coding process when resolving any coding disagreement.

## Results

### Participant Characteristics

Carer participant characteristics are summarized in Table 1. See Multimedia Appendix 5 for the breakdown of carer attendance at each focus group. We found 25.94% (12,507/48,211) of our total data set contained misconceptions or stigmatizing language originating from our negative search terms.

**Table 1:** Participant characteristics, N=7.

Characteristics			Values
**Gender, n (%)**			
	Female	5 (71)	
	Male	2 (29)	
Age (years), mean (SD)			63.33 (11.79)
**Ethnicity, n (%)**			
	White British	6 (86)	
	Black/Black British	1 (14)	
**Employment status, n^a^ (%)**			
	Retired	3 (50)	
	Employed (part-time)	1 (17)	
	Self-employed	1 (17)	
	Receiving Employment and Support Allowance (ESA)	1 (17)	
Length of time spent being a carer (years), mean (SD)^a^			8.83 (6.59)

^a^For this category, n=6 as there is 1 missing data point; percentages have been calculated accordingly.

### Carer Influence on the Framework (Focus Groups 1 to 3)

Carers’ feedback from focus groups 1 to 3 was used to construct 6 finalized categories: 3 neutral categories (lived experience, organizational and community group statements, and individual comments on dementia-related topics) and 3 negative categories (minimizing or underestimating words/statements; dehumanizing, weaponizing, or outdated words/statements; and incorrect or questionable words/statements).

### Final Tweet Categorization

In step 3, 6 carers categorized 250 tweets each, but 3 tweets were not categorized, leaving 1497 categorized tweets. See Table 2 for the number of tweets falling into each category.

There was fair agreement between carers across 6 categories (3 neutral and 3 negative) on average in the framework (*κ*=0.43; range 0.067-0.7). Agreement was better when we aggregated the data to investigate agreement between neutral and negative categories, but there was still evidence that carer views differed (*κ*=0.92; range 0.5-1).

**Table 2:** Carer attribution of tweets into each framework category (categories 1-3: neutral; categories 4-6: negative), n=1497.

Categories	Tweets categorized to each category, n (%)
1. Lived experience	97 (6.48)
2. Organizational and community group statements	308 (20.57)
3. Individual comments on dementia-related topics	232 (15.50)
4. Minimizing or underestimating words/statements	19 (1.27)
5. Dehumanizing, weaponizing, or outdated words/statements	662 (44.22)
6. Incorrect or questionable words/statements	96 (6.41)
7. Other^a^	34 (2.27)
8. I don’t know^a^	49 (3.27)

^a^For the purpose of categorization, 2 additional categories were created: other (for tweets that clearly did not belong in any of the other categories) and I don’t know (for tweets that carers thought might belong in one of the categories, but were uncertain about).

### Qualitative Analysis of Tweets

A total of 863 tweets were thematically analyzed from the 3 negative categories (minimizing or underestimating words/statements; dehumanizing, weaponizing, or outdated words/statements; and incorrect or questionable words/statements), as well as those categorized as “other” and “I don’t know.” All the coding discrepancies were resolved between the service user researchers. The summary of the final framework of themes is shown in Table 3 and Multimedia Appendix 6 with example tweets.

The majority of tweets were specifically insults targeted towards politicians (469/863, 54.3%), and a large portion contained general dehumanizing, weaponizing, or outdated words/statements (n=143, 16.6%). Dehumanizing language featured heavily in the tweets about politics (63/863, 7.3%), and the most frequently found words in the tweets featured American politicians alongside the words “senile” and “demented.”

**Table 3:** Carer defined framework categories, and their researcher defined themes and subthemes, showing the number of tweets coded to each theme and framework category and their percentage of the total number of tweets analyzed, n=863.

Framework categories, themes, and subthemes				Tweets, n (%)		Tweets coded to each framework category, n (%)
**Minimizing or underestimating words/statements**				1 (0.1)		21 (2.4)
	Jokes		14 (1.6)			
	Painting a negative picture		3 (0.3)			
	Unintentionally minimizing		3 (0.3)			
**Dehumanizing, weaponizing, or outdated words/statements**				143 (16.6)		737 (85.4)
	Celebrities		34 (3.9)			
	**Politics**		63 (7.3)			
		Weaponizing diagnoses	4 (0.5)			
		Insults targeted towards politicians	469 (54.3)			
	Unintentionally weaponizing		24 (2.8)			
**Incorrect/questionable words and statements**				0 (0)		34 (3.9)
	Armchair diagnoses		21 (2.4)			
	Cures/causes of dementia		11 (1.3)			
	Assumptions about politicians		2 (0.2)			
Neutral				64 (7.4)		64 (7.4)
Unclear				7 (0.8)		7 (0.8)

#### Minimizing or Underestimating Words/Statements

Tweets in this framework category made light of dementia, using nonoffensive words (eg, “selective dementia”) in a way that did not convey the seriousness of the condition. This was further nuanced by some tweets using dementia-related terms to make jokes about people’s unusual behavior or painting a negative picture of dementia. In these cases, tweets suggested that people with the condition have a poor quality of life, as if they are just waiting until “death ends your misery,” or are inherently a danger to themselves or others. Some tweets in this theme unintentionally minimized the severity of dementia, without using weaponizing language. These suggested that those diagnosed do not in fact have dementia, and elderly people should not be expected to “remember her relatives’ birthdays.”

#### Dehumanizing, Weaponizing, or Outdated Words/Statements

Tweets in this framework category used stigmatizing and weaponizing words to ridicule dementia or people with dementia, most frequently using “demented” or “senile.” The vast majority of these tweets were related to politics. Most were insults targeted towards politicians, most frequently Donald Trump (“Demented Don”) and Nancy Pelosi (“Nancy is a senile…woman”); however, Joe Biden also had many such insults targeted towards him (“Biden is senile”). Some tweets used weaponizing language casually to make weaponizing diagnoses of politicians (eg, tweeting that a politician “has senility”). The majority of these were about Donald Trump. Many tweets also referred to “demented democrats” generally. Tweets in this theme used this weaponizing language about celebrities, frequently Bette Midler. Some tweets used weaponizing terms unintentionally in reference to behaviors the user does not like, such as being “in bed before 11.30pm.”

#### Incorrect/Questionable Words and Statements

This framework category represents tweets that contained misconceptions around dementia. Most frequently, these took the form of armchair diagnoses, suggesting that somebody, likely a public figure, has dementia in a way that is not malicious. Most referenced Donald Trump; however, other politicians were also named, such as Bill Clinton, Ronald Reagan, and Joe Biden. Many used their personal experience of a client or relative’s dementia diagnosis as justification for their armchair diagnosis, reasoning that they have “lived with it with my Mom.” Additionally, these tweets speculated on causes of dementia, including “vegan diet and carbs,” or provided suggestions for cures that appeared anecdotal or were not supported by research findings.

#### Neutral

These tweets were judged by the researchers to not portray any negative attitudes towards dementia. One tweet referred to a film “Cecil B Demented,” with several others reporting on reputable scientific results in the field of dementia.

#### Unclear

This framework category contained tweets that the researchers could not categorize into other themes. Often, their meaning could vary depending on connotation, and it was unclear whether they were making light of dementia or legitimately referring to somebody with the condition (eg, “I thought he was brake checking me for a second but then I realized his dementia was effecting his motor skills”).

## Discussion

### Principal Findings

There is limited qualitative research investigating dementia misconceptions on Twitter, with most literature focusing on content relating to dementia awareness [12,20] or supporting people with dementia [13,21]. To our knowledge, this is the first participatory study focusing on dementia misconceptions on Twitter to develop a framework to categorize misconceptions. We found that dementia misconceptions and weaponizing terms are prevalent and problematic on Twitter.

From the tweets extracted on dementia, 25.94% (12,507/48,211) were negative. We then extracted a sample representing half negative and half neutral tweets and validated this categorization by carers’ ratings. They rated just over half of the tweets (777/1497, 51.90%) as displaying negative attitudes, which is slightly over the 50% (750/1500) of these tweets extracted using negative search terms. Most negative tweets were insults targeted towards politicians. Our prevalence of negative tweets (12,507/48,211, 25.94%,) is similar to previous work by Oscar et al [8], who found 21% of their Alzheimer disease–related tweets (N=6583) used Alzheimer disease–related words to perpetuate stigma. Their analysis was carried out by 2 researchers manually coding only 311 tweets across 6 broad categories (metaphorical, personal experience, informative, joke, ridicule, organization). Our participatory work focuses on the end-user views—the carers’ ratings and views of misconceptions. We found an overlapping theme in “jokes,” but through our qualitative analysis, we were able to highlight that jokes manifest as minimizing or underestimating words or statements. This high prevalence of misconceptions and stigma in tweets is mirrored in research investigating other neurological conditions. For example, McNeil et al [22] found 41% of tweets using the word “seizure” were derogatory in nature, and likewise found ridicule or jokes were common in these tweets. These misconceptions towards dementia are also widespread in the general population and are not exclusive to views disseminated on social media. Crisp et al [23] found that over half of the UK adults surveyed expressed negative attitudes towards people with dementia, including that they were unpredictable, hard to talk to, and feel things in a different way than other people.

We employed an inductive methodology to categorize each tweet into 1 theme. This approach has also been applied in previous qualitative research [12], but others adopted deductive approaches (with categories decided a priori) to categorize almost 70% of tweets to multiple dimensions [8]. We made the conscious decision to involve carers from the very beginning to develop a framework based on their experiences, and then employ an inductive approach for our qualitative analysis. This was important as this is the first piece of research to focus specifically on dementia misconceptions on Twitter, but it also ensured that we captured the meaning of the tweet from the recipient’s viewpoint (taking an emic perspective [24]), particularly given that tweets are short snippets of text which can lack context.

### Implications

This study has significant public health implications. We provide terms that carers of people with dementia consider to be misconceptions or stigmatizing towards dementia. Therefore, social media platforms should incorporate these terms into their algorithms to enable users to filter out any tweets containing these negative terms. As these terms have been generated by carers after conducting Twitter searches, their validity is reinforced as they have been rated as negative by the people they affect the most.

Additionally, these terms could be used to identify Twitter users who propagate these attitudes and target them in an awareness campaign to reduce their misconceptions. This would aim to promote awareness of the use of words which can perpetuate stigma around mental illness, benefiting the reduction of stigma related to any mental illness [25].

### Strengths and Limitations

Understanding what constitutes stigmatizing or weaponizing language on Twitter requires the incorporation of personal perspectives, but this approach is rare. Previous studies investigating misconceptions or stigma in mental health have rarely consulted with service users or carers [8,26,27]. Our participatory methods ensured that our framework is grounded in the personal perspective of those who will be affected by the poor use of language.

Our sample of tweets thematically analyzed by researchers (n=863) is larger than those in previous studies, such as Cheng et al [12] (n=398) and Oscar et al [8] (n=311), and this broader sample provides a better understanding of the prevalence and forms of dementia misconceptions on Twitter. However, many of our tweets were related to American politics; therefore, future work should consider using a broader time period to understand whether this effect is one of time (an election period) or one of American politics in general. The timing of tweet collection will have affected the prevalence of tweets relating to politicians and the rate may be lower if tweets are collected at other times.

Additionally, we extracted tweets during UK office hours and, therefore, overnight events would have been captured the following morning. This may not have allowed us to capture the initial conversations surrounding controversial events. This work only focuses on Twitter and Twitter users, who may not represent the general population [28] or users of other social media platforms. Future work should investigate misconceptions on other social media platforms and in the wider general public.

Our carer group was small and consisted predominately of White British participants, and there was mixed agreement by carers on what constitutes misconceptions and stigma. We found that agreement about tweet categories was greater when assessing whether a tweet was negative or neutral, rather than its individual category; some tweets could be interpreted as stigmatizing by one person, but not by another. Our findings reflect the heterogeneity in neurological and mental health conditions, combined with societal and cultural factors, which shape how individuals communicate and understand their mental health [29]. We propose that future work ensures not only a larger group, but also a more diverse group of carers, patients, and members of the public classify tweets, and that clinical, social, and cultural data are used to understand some of their personal reactions.

### Conclusion

This study demonstrates the importance of coproduction in assessing dementia misconceptions. Contributions from people with lived experience and carers can provide a perspective that may be overlooked by researchers. We highlight the high frequency of misconceptions or weaponizing language used in dementia-related tweets. The most commonly used terms are “demented” and “senile” to disparage American politicians including Nancy Pelosi, Donald Trump, and Joe Biden. These findings may prove to be useful to inform a campaign aiming to reduce these misconceptions, correct people’s misunderstandings of dementia, and highlight the effect their words have on carers of, and people with, dementia.

### Funding

This work was supported by the NIHR Biomedical Research Centre at South London and Maudsley NHS Foundation Trust and King’s College London (IS-BRC-1215-20018), and Alzheimer’s Research UK’s Inspire Fund awarded to SJ.

### Data Sharing

Data available upon request from SJ.

## Appendix

Multimedia Appendix 1 Number of tweets collected in each extraction round.

Multimedia Appendix 2 Breakdown of focus group content.

Multimedia Appendix 3 Preliminary themes with carer feedback .

Multimedia Appendix 4 Guidance document explaining coding as provided to third coder.

Multimedia Appendix 5 Breakdown of carer attendance by focus group.

Multimedia Appendix 6 Framework categories and themes with their subthemes, showing example tweets for each theme.

## Abbreviations

API: application programming interface
NHS: National Health Service
NIHR: National Institute for Health Research
URL: Uniform Resource Locator
