# Supplementary material for: Investigation of Carers’ Perspectives of Dementia Misconceptions on Twitter: Focus Group Study
Source: JMIR Aging. 2022 Jan 24;5(1):e30388. doi: 10.2196/30388 (PMC8822432; doi:10.2196/30388)
Supplement: Multimedia Appendix 1 [file aging_v5i1e30388_app1.docx]

| **Supplementary Table 1. Number of tweets collected in each extraction round** | | | | | | |
| --- | --- | --- | --- | --- | --- | --- |
| **Round** | **Dates of extraction** | **Times of extraction** | **Neutral search terms** | **Negative search terms** | |  |
| 1 | 04/02/2020 | 10:47 – 16:47 | 6068 | 1664 | |  |
| 2 | 05/02/2020 | 09:40 – 16:50 | 10537 | 4461 | |  |
| 3 | 06/02/2020 | 09:30 – 16:30 | 6323 | 2525 | |  |
| 4 | 07/02/2020 | 09:50 – 16:59 | 12776 | 3857 | |  |
| **Total** | | | **35704** | | **12507** | |
