# Supplementary material for: Investigation of Carers’ Perspectives of Dementia Misconceptions on Twitter: Focus Group Study
Source: JMIR Aging. 2022 Jan 24;5(1):e30388. doi: 10.2196/30388 (PMC8822432; doi:10.2196/30388)
Supplement: Multimedia Appendix 2 [file aging_v5i1e30388_app2.docx]

| **Supplementary Table 2. Breakdown of focus group content** | |
| --- | --- |
| **Focus group** | **Description** |
| 1 | Participants first discussed their first-hand experiences of conversations around dementia they have had in their lives with friends, healthcare professionals and members of the public, with a view to characterising the conversations that take place around dementia. Following the focus group, these discussions were transcribed and grouped into preliminary themes using content analysis (Erlingsson & Brysiewicz, 2017).  Participants also used Twitter to generate a list of search terms to return tweets focused on, or alluding to, dementia. These search terms were used to extract 48,211 tweets which were subsequently refined to 2,000 usable tweets. |
| 2 | Participants provided feedback on the preliminary themes. Subsequently, participants collectively categorised 250 tweets into the preliminary themes to test how well they characterised and fitted Twitter conversations around dementia. This was done with the purpose of providing further feedback to refine the preliminary themes. |
| 3 | Participants collectively categorised a further 250 tweets into the preliminary themes to test their applicability to Twitter conversations around dementia. Participants subsequently provided further feedback on the preliminary themes to improve the extent to which they characterised Twitter conversations around dementia. After the focus group, feedback from focus groups 2 and 3 was used by the research team to produce 6 finalised themes. |
