# Supplementary material for: Investigation of Carers’ Perspectives of Dementia Misconceptions on Twitter: Focus Group Study
Source: JMIR Aging. 2022 Jan 24;5(1):e30388. doi: 10.2196/30388 (PMC8822432; doi:10.2196/30388)
Supplement: Multimedia Appendix 3 [file aging_v5i1e30388_app3.docx]

**Multimedia Appendix 3.** Preliminary themes with carer feedback**.**

**Preliminary themes (Focus group 1-3)**

**Useful advice for carers to improve their experience or the experience of those they are caring for**

Pre-initial tweet categorisation

Participants discussed general advice and the experience of those with dementia, including how to make the situation more comfortable for the person experiencing the condition, as well as advice they would have liked to know earlier on.

*Advice that I'd never received would have been to restrict television viewing: things like Coronation Street are designed to wind you up; they create emotion, and emotion was the very, very last thing my mum needed.*

Post-initial tweet initial categorisation

Overall, it appeared participants were not able to identify many tweets that fit into this category, in particular as they felt it difficult to gauge whether the advice given could be seen as useful. Nevertheless, some tweets contained lists with useful activities for people with dementia.

**Gratitude for support that currently exists**

Pre-initial tweet categorisation

Participants talked about their appreciation for currently available support, such as specialist services in the NHS, support groups, supportive clinicians, useful aids, adaptations to the home environment and the fact that support is easier to access nowadays.

*We have a local support group where we were all in our 50s when our husbands developed dementia. At difficult times the group was a wonderful understanding source of support and now we are a social group for each other.*

However, participants also highlighted the issue of lack of awareness of such support.

*Often people aren't aware of what is available in their area, and often what is available is hugely provided by volunteers rather than the public purse.*

Post-initial tweet categorisation

Participants did not find many tweets that fit into this category, questioning whether it was in fact needed and if so, the title should be changed into “Information about support available”, as most tweets contained information about raising awareness.

**Factors that inspire a sense of hope, or a feeling that things will get better in the future**

Pre-initial tweet categorisation

Some comments made by participants indicated a sense of hope that things would improve, in particular due to the progression of dementia research and the ways in which taking part in research themselves made them feel that they had made a positive contribution.

*But they leave thinking 'well I've done something positive today', and they brought such strength from that, and participating in research, I'm involved in a heck of a lot of research.*

One participant also stated that they felt dementia stigma had reduced and would continue to, in the way that stigma around cancer had.

*No one talks about the 'Big C' any longer, people don't talk in hushed tones about dementia - people talk openly nowadays about dementia.*

However, another participant did not identify with the theme, commenting that they felt research was too long-term.

*For me, research seems very long-term, and not something that's going to help in the here-and-now. I'm involved in the research because I want people in the future to have other options, but I'm not imagining that it may be in even my lifetime(…) It's more like my intent to make a different future, or my intent to make a difference, maybe for the next or following generations. But a sense of hope - I'm not sure I had that even from my participation.*

Post initial-tweet categorisation

Participants found very few tweets that could belong to this theme and wondered if this could be used as an opportunity to encourage such conversations online. Furthermore, participants found it difficult to assess whether factual tweets belonged into this category.

**Minimising or underestimating the experience of living with or supporting someone with dementia**

Pre-initial tweet categorisation

Participants commented on ways in which other people tended to trivialise the condition, often as a result of being ill-informed, as well as the lack of support offered by members of the public.

*People who look on but don’t support you when you are struggling e.g. to guide the person you are caring for to sit down on the bus.*

However, participants viewed this theme as an opportunity to educate people.

*(…) the positive side of this is that it's a means of educating people. It's the contrast between what somebody thinks as an outsider: 'oh well, all they've got to do is that', and what is the reality. So the education would be filling that gap in some way between what someone who has no experience of it thinks is relatively easy/relatively not a burden, and the reality. So it is a means of educating that group.”*

Post-initial tweet categorisation

Participants found a lot of overlap between theme 4 and 5 and were unsure at times whether the tweet came from an external or direct experience. One participant found very few tweets in relation to this theme but noted that it is still important to educate people on this issue.

**Dehumanising and stigmatising language, conversations, and experiences.**

Pre-initial tweet categorisation

Participants discussed conversations they had with other people, during which words were used and statements were made that indicated a lack of regard for the individual with dementia, a trivialisation of the reality of the condition and an infantilisation the person.

*My neighbour said it’s like having a child.*

In addition, participants commented on experiences that they felt dehumanised not only the person with dementia, but also the carer.

*Also the detail they go into your finances if you ask for any assistance - I think we had to get 10 years of bank statements, and they query every outgoing, so it's quite intrusive. I felt dehumanised by social services, people who were supposedly helping - they made my life a misery.*

Post-initial tweet categorisation

Participants found many tweets relating to this category and commented on the importance of addressing the issue of the use of stigmatising words, such as “senile” and “demented”. However, one person questioned whether language should be censored.
