# Supplementary material for: Investigation of Carers’ Perspectives of Dementia Misconceptions on Twitter: Focus Group Study
Source: JMIR Aging. 2022 Jan 24;5(1):e30388. doi: 10.2196/30388 (PMC8822432; doi:10.2196/30388)
Supplement: Multimedia Appendix 4 [file aging_v5i1e30388_app4.docx]

| **Supplementary Table 4.** Guidance document explaining coding as provided to third coder. | |
| --- | --- |
| **Framework Category**, Theme, and S*ub-theme* | **Description** |
| 1. **Minimising or Underestimating Words / Statements** | Making light of dementia or minimising it. |
| 1.1 Jokes | Making fun of people with dementia/the condition itself |
| 1.2 Painting a Negative Picture | Painting an overly negative picture of dementia |
| - 1. Unintentionally Minimising | Tweets that weaponise dementia (but not using stigmatising words) to make light of/ minimise the condition, at the expense of someone else, yet not intending to do so |
| 1. **Dehumanising, weaponising, or outdated words / statements** | Tweets that use weaponising or stigmatising words, like senile/demented/etc, targeted at public figures (excluding politicians) |
| 2.1 Celebrities | Tweets that use weaponising or stigmatising words, like senile/demented/etc, targeted at public figures (excluding politicians) |
| 2.2 Politics | Using stigmatising language in the context of politics, not directed at an individual but groups or situations (e.g. to insult members of a political party) |
| *2.2.1 Weaponising diagnoses* | Using stigmatising language to “diagnose” politicians with dementia. This is more of an explicit attempt at diagnosis, rather than just saying someone is “demented” etc. |
| *2.2.2 Insults targeted towards*  *politicians* | Using stigmatising language to insult politicians specifically |
| - 1. Unintentionally Weaponising | Using stigmatising language but not with the intention of weaponising/insulting. Usually in a self-deprecation context or used as slang to describe a negative situation |
| 1. **Incorrect / Questionable Words and Statements** | Questionable or incorrect statements about dementia and the way the condition affects people |
| 3.1 Armchair Diagnoses | Diagnosing people with dementia, without using stigmatising language. |
| 3.2 Cures / Causes of Dementia | Suggesting cures and causes of dementia without reference to research, usually based on anecdotal evidence or pseudoscience |
| 3.3 Assumptions about Politicians | Making assumptions about dementia in the context of politics |
| 1. **Neutral** | Tweets we believe to be neutral, they are not using stigmatising language, minimising/making light of dementia or making questionable statements |
| 1. **Unclear** | Tweets we were not sure about, as it depends on the context and tone used. |
