# Supplementary material for: Investigation of Carers’ Perspectives of Dementia Misconceptions on Twitter: Focus Group Study
Source: JMIR Aging. 2022 Jan 24;5(1):e30388. doi: 10.2196/30388 (PMC8822432; doi:10.2196/30388)
Supplement: Multimedia Appendix 5 [file aging_v5i1e30388_app5.docx]

| **Supplementary Table 5.** Breakdown of carer attendance by focus group | | | | |
| --- | --- | --- | --- | --- |
| **Carer ID** | **Focus Group 1** | **Focus Group 2** | **Focus Group 3** | **Rating Final Tweets** |
| **01** | ✓ | ✓ | ✓ | 🗴 |
| **02** | ✓ | ✓ | ✓ | ✓ |
| **03** | ✓ | ✓ | ✓ | ✓ |
| **04** | ✓ | 🗴 | ✓ | ✓ |
| **05** | 🗴 | ✓ | ✓ | ✓ |
| **06** | 🗴 | ✓ | 🗴 | ✓ |
| **07** | 🗴 | 🗴 | 🗴 | ✓ |
| **Total n** | **4** | **5** | **5** | **6** |
