# Supplementary material for: Investigation of Carers’ Perspectives of Dementia Misconceptions on Twitter: Focus Group Study
Source: JMIR Aging. 2022 Jan 24;5(1):e30388. doi: 10.2196/30388 (PMC8822432; doi:10.2196/30388)
Supplement: Multimedia Appendix 6 [file aging_v5i1e30388_app6.docx]

| **Supplementary Table 6.** Framework categories and themes with their subthemes, showing example tweets for each theme. | |
| --- | --- |
| **Framework Category**, Theme, and S*ub-theme* | **Example Tweet** |
| 1. **Minimising or Underestimating Words / Statements** | *“I work at a rehab hospital Monday-Saturday. 3-4hrs in the morning & a pain management clinic Monday-Friday evenings. I want to work at a dementia home or something PRN :\ I legit love what I do lol it’s so crazy. I’m always at work* 😂*”* |
| 1.1 Jokes | *“My grandpa just referred to tortillas as "fajita bread" and I know that sounds like senility, but that's literally the kind of shit you say after growing up in Franklin County Virginia”* |
| 1.2 Painting a Negative Picture | *“Yeah man just chilling in the nursing home having a brew fell over instantly passed away aneurysm I think be glad he was in for years getting worse with dementia etc only been in 3 days mad”.* |
| - 1. Unintentionally Minimising | *“can't believe all these senior citizens are truthfully diagnosed with dementia, some seem ok to me, lots of history to remember, should not have to remember her relatives birthdays. Younger relatives should remember their birthday”* |
| 1. **Dehumanising, weaponising, or outdated words / statements** | *“Someone needs to check on her. She's demented."* |
| 2.1 Celebrities | *“@BetteMidler Its a joke u crazy bitch!  unbelievable how demented u have gotten in your old age u don’t have a relationship with reality”* |
| 2.2 Politics | *“Why is it that democrats either look demented, like they’re on serious meds or like doofuses?”*  *“Another demented leftie ROFL* 🤣*”* |
| *2.2.1 Weaponising Diagnoses* | *“People with senile dementia become irrationally paranoid. Trump is a very dangerous man”* |
| *2.2.2 Insults targeted towards politicians* | *“DEMENTED is a word that fits Trump perfectly !!!”* |
| - 1. Unintentionally Weaponising | *“Beauty Tips * deconstruct/reinvent the idea of beauty as a fixed state on a daily basis *nurture + cultivate joy for no reason, 4 the pure joy of it *masturbate + drop the shame *Spread out, be comfy + at home in your own skin, as ugly as it may be according to demented standards”* |
| 1. **Incorrect / Questionable Words and Statements** |  |
| 3.1 Armchair Diagnoses | *“I am! Not kidding. I’ve lived it with my Mom. This lady is clearly showing signs of dementia!”* |
| 3.2 Cures / Causes of Dementia | *“Having that “Dad Bod” dramatically increases your risk for dementia! Currently, 5 years of dementia care costs around $300,000 and that price is going up!! The #CarnivoreDiet is a tremendous tool to get you lean...”* |
| 3.3 Assumptions about politicians | *“It isn't DEMENTIA. It is years of addiction. Everyone know we have an #AddictPresident. @GOP doesn't care. They are watching him break laws. They are making money. They know he is high, he sh*ts himself, creates havoc for more (not Sundowning). He's brain addled, mind glitching.”* |
| 1. **Neutral** | *“There are currently around 850,000 people with dementia in the UK. This is expected to rise to 1.6 million by 2040.”* |
| 1. **Unclear** | *“I thought he was brake checking me for a second but then I realized his dementia was effecting his motor skills”* |
